# The Koch Secret Announced

**Published:** 1891-02

**Authors:** 


BUFFALO MEDICAL AND SURGICAL JOURNAL

A MONTHLY REVIEW OF MEDICINE AND SURGERY.

*EDITORS:*

THOMAS LOTHROP, M. D. - - WM. WARREN POTTER, M. D.

All communications, whether of a literary or business character, should be addressed to the editors : 284 Franklin Street, Buffalo, N. Y.

Vol. XXX. FEBRUARY, 1891. No. 7.

### Editorial.

### THE KOCH SECRET ANNOUNCED.

Last week (Jan. 15th) the press despatches contained the purport of Professor Koch's communication, revealing all he then himself knew about the mysterious liquid that has made his name a household word during the last few weeks.

According to this account the material that has been expected to accomplish so much for the wasted consumptive, consists of an extract from a pure culture of the tubercle bacillus, by means of a forty to fifty per cent. dilution of glycerine. Professor Koch does not know, so he announces, just what the nature of this active principle is, but he proceeds to detail the methods by which he reached his conclusions as to its efficiency in the arrest of tuberculous processes. He says if a healthy guinea-pig be inoculated with the pure cultivation of German culture of tubercle bacilli, the wound closes over with a sticky matter and appears to heal promptly. After ten to fourteen days a hard nodule presents itself which, soon breaking, forms an ulcerating sore, which continues until the animal dies.

On the other hand, if a tuberculous guinea-pig is inoculated, the first or second day thereafter the place of injection becomes hard, assumes a dark color, which spreads to the neighboring parts, attaining, usually, a diameter of .05 to 1 centimeter. In a few days more the skin becomes necrotic and finally falls off, leaving a flat ulceration, which usually heals rapidly and permanently.

We now quote at length from the communication itself :

Thus the injected tubercular bacilli quite differently affect the skin of a healthy guinea-pig from one affected with tuberculosis. This effect is not exclusively produced with living tubercular bacilli, but is also observed with the dead bacilli, the result being the same whether, as I discovered by experiments at the outset, the bacilli are killed by a somewhat prolonged application of a low temperature or boiling heat, or by means of certain chemicals.

This peculiar fact I followed up in all directions and this further result was obtained, that killed pure cultivations of tubercular bacilli, after rinsing in water, might be injected in great quantities under a healthy guinea-pig's skin without anything occurring beyond local suppuration. Tuberculous guinea-pigs on the other hand are killed by the injection of very small quantities of such diluted cultivations. In fact, within six to forty-eight hours, according to the strength of the dose, an injection which is not sufficient to produce the death of the animal may cause extended necrosis of the skin in the vicinity of the place of injection.

If the dilution is still further diluted until it is scarcely visibly clouded, the animals inoculated remain alive, and a noticeable improvement in their condition soon supervenes. If the injections are continued at intervals of from one to two days, the ulcerated inoculation wound becomes smaller and finally scars over, which otherwise it never does; the size of the swollen lymphatic glands is reduced, the body becomes better nourished, and the morbid process ceases, unless it has gone too far, in which case the animal perishes from exhaustion.

By this means the basis of curative process against tuberculosis was established. Against the practical application of such dilutions of dead tubercle bacilli, there presented itself the fact that the tubercle bacilli are not absorbed at the inoculation points, nor do they disappear in another way, but for a long time remain unchanged and engender greater or smaller suppurative food. Anything, therefore, intended to exercise a healing effect on the tuberculous process, must be a soluble substance which would be lixiviated to a certain extent by the fluids of the body floating around the tubercle bacilli and be transferred in a fairly-rapid manner to the pieces of the body, while the substance producing suppuration apparently remains behind in ths tubercular bacilli or dissolves but very slowly.

The only important point was, therefore, to induce outside the body the process going on inside if possible, and to extract from the tubercular bacilli alone the curative substance. This demanded time and toil until I finally succeeded, with the aid of a forty to fifty-per cent. solution of glycerine, in obtaining an effective substance from the tubercular bacilli. With the fluid so obtained I made further experiments on animals and finally on human beings. These fluids were given to other physicians to enable them to repeat the experiments.

Professor Koch thinks the experiments made by others bear him out in his claims, viz., that the remedy has a specific effect on the tubercular tissues, and is, therefore, applicable as a very delicate and sure reagent for discovering latent, and diagnosticating tuberculous processes.

The lay press, with its usual scientific acumen, has pronounced the Koch discovery a failure; whereas, the opinion of those who know most about it feel that it has a certain not yet well-defined field of usefulness, and that time and further experimentation are necessary to an intelligent understanding of the capabilities and limitations of Professor Koch's discovery. Let us wait patiently.

---

ERRATA.— The following corrections of typographical errors may be made in the JOURNAL for December, 1890, page 287 : Line 10 from above, leave out "aid ;" line 12 from above, for "temporarily," read temporally ; line 24 from above, for "consideration," read connection.

---

EIGHT patients are being treated in the Post-Graduate Hospital by Koch's lymph. Three of them are cases of lupus ; four are cases of phthisis pulmonalis, and one laryngeal tuberculosis. The inoculations are in charge of Dr. W. C. Bailey, who was for a long time a student in Koch's laboratory, assisted by the director of the laboratory, Dr. J. H. Linsley.

